# Ozone treatment of recurrent aphthous stomatitis: a double blinded study

**DOI:** 10.1038/srep27772

**Published:** 2016-06-15

**Authors:** Mahmoud K. AL-Omiri, Mohannad Alhijawi, Bader K. AlZarea, Ra’ed S. Abul Hassan, Edward Lynch

**Affiliations:** 1 Faculty of Dentistry, University of Jordan, Amman, Jordan; 2 The City of London School of Dentistry, BPP University, UK; 3 Department of Dentistry, Ministry of Health, Amman, Jordan; 4 Faculty of Dentistry, Aljouf University, Sakaka, Saudi Arabia; 5 Faculty of Allied Medical Sciences, The Royal University for Medical Sciences, Amman, Jordan; 6 Dentistry, Warwick Medical School, Coventry, UK

## Abstract

This study aimed to evaluate the use of ozone to treat recurrent aphthous stomatitis (RAS). Consecutive sixty-nine participants with RAS were recruited into this non-randomized double blind, controlled cohort observational study (test group). A control group of 69 RAS patients who matched test group with age and gender was recruited. RAS lesions in test group were exposed to ozone in air for 60 seconds while controls received only air. Ulcer size and pain were recorded for each participant at baseline and daily for 15 days. Ulcer duration was determined by recording the time taken for ulcers to disappear. The main outcome measures were pain due to the ulcer, ulcer size and ulcer duration. 138 RAS participants (69 participants and 69 controls) were analyzed. Ulcer size was reduced starting from the second day in test group and from the fourth day in controls (p ≤ 0.004). Pain levels were reduced starting from the first day in the test group and from the third day in controls (p ≤ 0.001). Ulcer duration, ulcer size after day 2 and pain levels were more reduced in the test group. In conclusion, application of ozone on RAS lesions for 60 seconds reduced pain levels and enhanced ulcers’ healing by reducing ulcers’ size and duration.

Ozone (O3) therapy has gained a lot of attention in medicine and dentistry because it is a powerful oxidant, has strong antimicrobial activity (against bacteria, viruses, yeasts and protozoa), can stimulate blood circulation and the immune response, and has analgesic effects^1^,^2^. However, respiratory tract mucosal cell lining is sensitive to oxidation; therefore, ozone is an irritant to respiratory system and can induce and promote allergy and asthma if inhaled directly in high doses. This underlines the importance of using adequate delivery systems to prevent ozone leakage and avoid its hazards to the respiratory system^1^,^2^,^3^,^4^,^5^.

Ozone has been used to treat about 250 different pathologies^1^,^2^,^3^,^4^,^5^. Dental applications of ozone included prevention and treatment of dental caries^6^,^7^,^8^,^9^,^10^,^11^,^12^, tooth remineralization^3^,^4^,^5^,^6^,^7^,^8^,^9^,^10^,^11^,^12^, infection control^1^,^2^,^3^,^4^,^5^,^6^,^13^, disinfection of periodontal pockets^14^, tooth bleaching^15^, pain control^1^,^2^,^3^,^4^,^5^,^14^, management of exposed roots and tooth sensitivity^3^,^4^,^5^, TMJ treatment^3^,^4^,^5^, endodontic treatment^3^,^4^,^5^, biofilm purging^1^,^2^,^3^,^4^,^5^, acceleration of healing^3^,^4^,^5^, tissue regeneration^2^,^3^,^4^,^5^,^6^,^7^,^8^,^9^,^10^,^11^,^12^ and control of halitosis^3^,^4^,^5^.

Recurrent aphthous stomatitis (RAS) is a well known disorder that affects the oral mucosa and is distinguished by recurrent, single or multiple, painful, round or oval ulcers with erythematous borders^16^,^17^. It negatively impacts quality of life^18^,^19^. Many treatment paradigms have been used for management of oral ulcers including mouthrinses (such as chlorhexidine), topical corticosteroids, topical tetracycline, immunoregulators (such as levamisole, azathioprine and colchicine), TNF inhibitors, systemic zinc sulphate, monoamine-oxidase inhibitors, sodium cromoglycate, deglycirrhizinated liquorice, sucralphate etretinate, low-energy laser, herbs and others. Unfortunately, no specific cure for RAS is available. On the other hand, available treatments might reduce RAS symptoms but cannot totally cure RAS or prevent ulcer recurrences^16^,^17^.

Ozone has been used for the treatment of ulcers affecting other parts of the gastrointestinal tract^20^,^21^,^22^, blood vessels^23^ and skin^24^,^25^; however, none have studied the role of ozone in treatment of recurrent aphthous ulcers in the oral cavity.

This motivated the conduction of this study in order to assess the role of ozone in management of recurrent aphthous ulcers.

The aim of this study was to assess the therapeutic effects of 60 seconds of ozone application on ulcer size, ulcer duration and pain caused by recurrent aphthous ulcers.

The null hypothesis for this study was that ozone application for 60 seconds has no role in treatment of RAS lesions and has no effects on ulcer size, ulcer duration or pain caused by RAS lesions.

## Materials and Methods

Sixty nine consecutive patients (30 men and 39 women) aged between 18 and 40 years old (mean age  ±  SD: 27.2  ± 7.5 years) were recruited into this parallel, controlled, double blinded study. The study was conducted during the period from 1^st^ of February until 30^th^ November 2015. The participants were diagnosed with RAS and were visiting dental clinics at the University of Jordan, Amman, Jordan.

This study was carried out following ethical principles, including the World Medical Association Declaration of Helsinki. The study was ethically approved by the Deanship of Research, University of Jordan (Reference number 15-2015). All participants were provided with a full explanation of the study, and participants’ informed consent was obtained before participation in the study.

In order to be included in the study, participants’ age ranged from 18 to 40 years old in order to avoid the potential effects of age on healing of lesions. Also, they had no diagnosed medical disease (including previous mental or psychological disorders, auto-inflammatory disorders, hormonal conditions, haematinic deficiencies, Behcet disease, diabetes, cardiovascular, hypertension, gastrointestinal, skin, liver or renal disease)^16^,^17^,^18^,^19^. In addition, participants had intraoral labial ulcers, received no treatment for the ulcers before being included in the study and had no active periodontitis. Participants had to present for the purpose of this study on the first day of ulcer appearance.

Participants with removable prosthetic rehabilitations or orthodontic appliances were excluded from the study to avoid potential effects of the appliances on healing of the lesions. Also, participants who chewed tobacco, smoked cigarettes or smoked Narghile (water pipe) were excluded from the study. Also, participants were excluded if they had received any treatment for their ulcers before inclusion in the study.

Medical assessment of each participant included detailed medical and dental histories, complaints, personal information (name, age, gender, occupation, education, marital status and address), full clinical examination, and selected laboratory tests (complete blood count, red blood cell folate, serum vitamin B12, and serum ferritin) were carried out for differential diagnosis and assisting exclusion of underlying medical disease following the methodology of previous literature^16^,^17^,^18^,^19^.

Ulcers were regarded as RAS when they were grey or yellowish in colour, surrounded by an erythematous margin, painful, recurrent and not associated with systemic disease^16^,^17^,^18^,^19^. All clinical examinations were carried out on a dental chair set with a light unit Daray angle poise extra-oral light source (Daray Lighting Ltd, Leighton Buzzard, and Bedfordshire, UK). Dental oral mirrors (15/16 inch, Hanhnenkratt GMBH, Germany), explorer probes (0700-9 anatomical handle single ended, ASA Dental Co, Italy) and periodontal probes were utilized during the intra-oral examinations.

Main study outcome measures were pain levels due to ulcers, size of ulcers and duration of ulcers. Ulcer size was recorded at study baseline using a digital caliber (Terensa, USA) which has an accuracy up to 0.01 mm. Also, pain assessment was carried out at study baseline using a VAS score recorded from 0 to 10; 0 meant no pain and 10 meant the most severe pain. Then, the ulcers in the test group were exposed to 2350 ppm of ozone gas with a flow rate of 615 cc per minute for 60 seconds using the healOzone X4 machine (healOzone X4, Curozone, Germany) which required an adequate seal of its delivery system to be able to work and provide ozone^8^,^14^. An ozone measurement device was used to confirm the ppm of ozone delivered and a flow meter was used to confirm the flow rate immediately before the start of the study. Ozone was applied on the lesions through special disposable silicone cups that permitted adequate seal and avoided gas escape which ensured the safety of the machine for human use^8^,^14^. No ozone could escape and therefore no ozone smell could be detected which allowed blinding. The large cup with an 8 mm diameter was used on each occasion and when the ulcer was larger than 8 mms then the cup application was repeated so that the entire area of the ulcer was treated with ozone. Ulcer size and pain assessment were then repeated as above daily for 15 days. Ulcers duration was determined by recording the time (in days) required for the ulcers to disappear.

Each participant was examined on a dental chair following the above described procedures everyday throughout the study to also rule out any other changes in their oral and dental status apart from the assessed ulcers (such as having new orthodontic or prosthetic appliances, having dental pain due to other reasons, or having trauma to the mucosa or teeth). Subjects would have been excluded if they had any such changes to avoid any potential effects of such changes on ulcer healing as well as assessment of pain and ulcer size and duration. The participants were instructed not to use any systemic or local drugs for the treatment of the ulcers or their symptoms throughout the period of the study. They were to be excluded from the study if they used any other therapy for the ulcers. In this study, all participants completed the study without any drop out (drop out rate was zero).

A control group of 69 RAS patients (39 women and 30 men, mean age  ±  SD: 27.4 ± 7.1 years) who matched the participants in the main study group with age and gender were also recruited into this study. The inclusion and exclusion criteria and medical assessment of the controls were the same as those followed during the recruitment of participants in the test group. Pain assessment as well as recording ulcers size was carried out at study baseline following the same procedures as in the test group. The used healOzone machine was specially designed by having a switch at the back of the device which was switched to deliver only air and no ozone for the controls. Participants in the control group received the same treatment but with air only with no ozone and no participant was informed if they were in the test or the control group. The controls were also followed up every day for 15 days. Ulcer size and pain assessment were repeated daily for 15 days following the same procedures in the test group. Ulcer duration was determined by recording the time required for the ulcer to disappear.

One examiner blindly conducted all clinical examinations and intra-examiner reliability was evaluated on eight duplicate clinical examinations utilizing Kappa statistics. The Kappa value was 0.91 signifying a high agreement. Also, inter-examiner reliability was evaluated by conducting the same eight examinations by another investigator; Kappa was 0.87 demonstrating high agreement. Neither the examiner nor the participants were aware of which participant received ozone or only air.

### Statistical analysis

The data analysis was executed using the SPSS computer software (Statistical Package for the Social Sciences; version 19.0, SPSS Inc.). The Pearson Correlation test was utilized to test for correlation between participants’ demographic and pain levels, ulcer size and ulcer duration. A paired sample t-test was utilized to evaluate pain levels and ulcer size before and after ozone application in the main study group. The ANOVA test was used compare pain levels, ulcer size and ulcer duration between groups. Statistical significance levels were set at P  ≤  0.05 during all statistical analyses. The sample size for this study was estimated via the software G*Power 3.1.9.2 (Heinrich-Heine-University Dusseldorf, Dusseldorf, Germany) by utilizing data obtained following a pilot study. The repeated measurements design was used in the software, in which the ozone treatment was considered the independent factor and the ulcers size as the repeated measures factor. With a significant level of 5%, statistic power of 80%, and effect size of 20%, the minimum sample size was calculated as 52 participants for each group.

## Results

RAS participants (69 participants and 69 controls) were included in the analysis. Ulcer duration in the test group ranged between 7 and 10 days (mean  ±  SD: 8.4 ± 0.8 days). On the other hand, ulcer duration in the control group ranged between 8 and 12 days (mean  ±  SD: 9.5 ± 0.6 days).

Table 1 presents the distribution of ulcer size among study groups at baseline and daily for 15 days. Ulcer size was reduced as time progressed in both groups; however, reduction in ulcer size was more pronounced in the test group where ozone was applied on RAS lesions.

Table 2 shows the distribution of VAS scores for pain levels among study groups at baseline and daily for 15 days. VAS scores for pain in the test group were reduced after application of ozone, and were reduced with time more than those in the control group.

Correlations within the test group showed that no significant relationships were found between age and gender on one side and each of ulcer duration, ulcer size, and pain levels (p > 0.05) (Table 3). On the other hand, within the control group; no significant relationships were found between age and each of ulcer duration, ulcer size and pain levels (p > 0.05) (Table 3). Also, no significant relationships were found between gender and each of ulcer size and pain levels among controls (p > 0.05). However, males had a lower ulcer duration than females (p = 0.020) (Table 3).

Furthermore, paired samples t-test showed that ulcer size was significantly reduced starting from the second day following ozone application (p ≤ 0.002) and starting from the fourth day among controls (p ≤ 0.004) (Table 4). Also, paired samples t-test showed that pain levels were significantly reduced starting from the first day shortly following ozone application (p ≤ 0.001) and starting from the third day among controls (p ≤ 0.001) (Table 5).

Comparison between groups using the ANOVA test showed that there were no significant differences between groups regarding participants’ age, participants’ gender, ulcer size at study baseline, ulcer size at day 2, and pain levels at baseline (p > 0.05).

On the other hand, ulcer duration, ulcer size from day 3 untill day 10, and pain levels from day 1 untill day 10 were more reduced in the test group treated with ozone in comparison to controls (p ≤ 0.012).

## Discussion

The findings of this study demonstrated that application of ozone for 60 seconds on RAS lesions resulted in lower pain levels and improved healing of ulcers. Therefore, the null hypothesis of the study was rejected.

The healOzone X4 machine was utilized to produce and deliver ozone in this study because it was employed in many clinical trials previously and documented to be safe owing to the total confinement of ozone within the machines delivery system^8^,^12^,^13^,^15^. Miller and Hodson^8^ established that the Ozicure machine caused ozone accumulation to unsafe levels for humans typically if operated without adequate suction; and thus should not be used. Alternatively, the healOzone machine was confirmed safe to use since it is designed to function only if the delivering cups provide an adequate seal to avoid ozone leakage^8^,^12^,^13^,^15^.

In this study, ozone was directly applied to oral mucosa and did not cause any negative tissue reaction. This harmonizes with the findings of previous studies which concluded that ozone had less toxic effects on human oral epithelium and gingival fibroblast cells than chlorhexidine, sodium hypochlorite or hydrogen peroxide^26^. Also, Hauser-Gerspach *et al*.^27^ found that ozone did not affect osteoblastic cell adhesion and proliferation. In addition, previous studies applied ozone directly on teeth, soft periodontal tissues, gingiva, oral mucosa, bone and periimplant tissues and no adverse events were recorded in any study^3^,^4^,^5^,^6^,^12^,^13^,^26^,^27^,^28^,^29^,^30^,^31^,^32^.

The findings of this study demonstrated that pain levels were significantly reduced following ozone application on RAS ulcers. This concurs with the findings of previous studies that applied ozone to soft tissues and bone during third molar extraction and revealed that ozone application was associated with reduction in pain levels^28^,^30^,^31^. Also, this supports the hypothesis that ozone is associated with analgesic effects^1^,^2^,^3^,^4^,^5^.

The results of this study showed that ulcers healing duration as well as ulcers size were significantly reduced following the application of ozone. This might be explained by ozone properties including being a powerful oxidant and ability to stimulate blood circulation and immune responses^1^,^2^,^3^,^4^,^5^. An inflammatory cell infiltrate (including neutrophils and lymphocytes) exists beneath ulcers and produces inflammatory mediators that are responsible for the inflammatory process seen in RAS lesions. Being a powerful oxidant; ozone affects both the cellular and humoral immune system, oxidizes toxins making their excretion easier, encourages production of immunocompetent cells and immunoglobulins, improves phagocytosis function of macrophages, and induces production of tumor necrotizing factor (TNF-α), leukotrienes, interleukins, and prostaglandins that ends inflammation and accelerate tissue healing^33^. Furthermore, ozone enhances the oxygen carrying capacity of blood leading to more efficient cellular metabolism of inflamed tissues and better utilization of energy via activation of aerobic pathways of metabolism (Krebs cycle, glycolysis, and β-oxidation of fatty acids). In addition, oxidant activity of ozone initiates protein synthesis and enhances cellular ribosomes and mitochondria. Consequently, cellular activity and regeneration potentials will be improved and boost tissue healing^33^.

Such properties might potentially accelerate healing of the lesions leading to reductions of ulcer size and ulcer duration. This agreed with previous studies that applied ozone for the treatment of other types of ulcers (such as ulcers involving the stomach, duodenum, veins, and skin) and revealed that ozone was associated with better healing of the ulcers^20^,^21^,^22^,^23^,^24^,^25^. However, this contrasts with the findings of previous studies that applied ozone to bone and soft tissues during third molar surgery and found that it did not affect healing time but significantly reduced pain levels^28^,^30^,^31^. This contrast could be attributable to differences in sample size and study design such as the utilization of another machine that generated dramatically lower ozone concentrations and lower flow rates with a much lower delivered dose of ozone, application of ozone for shorter time periods and applying ozone to different tissues during bone surgery.

This study demonstrated that ozone application improved healing of ulcers and reduced pain.

Therefore, ozone should be considered for the management of RAS lesions as it can reduce treatment time, reduce oral ulcers and impacts on quality of life by reducing pain and ulcer duration, and thus improve patients’ perception of and compliance with treatment. Also, application of ozone is convenient, fast and monitored since it is administered by this special machine that can control concentration, volume, application site and timing of ozone application.

The study limitations are that the size of the study sample is small; nevertheless, the sample size in this study is comparable to or even larger than the sample size in previous investigations in this field^15^,^18^,^19^,^20^,^21^,^22^,^23^,^24^,^25^. Also, pain assessment in this study depended on subjective reporting of patients’ opinions regarding pain levels. This is an inherent drawback in subjective and self reporting of symptoms. However, all attempts were made to provide patients with a full explanation of the VAS scale used to score pain levels in this study. Another limitation of using ozone as a therapy for RAS patients is that there is no home device for patients to use, and that some patients would have very frequent RAS attacks and would not be able to attend the clinic each time they have an attack. Current studies are in progress to investigate the effects of ozone therapy on frequency of RAS attacks as well as to test new methods for ozone administration schedules and dosage that can be applied by patients at home. It would be useful to establish whether changes in ozone dosage or schedule of administration might achieve more impressive answers for patients in future.

Further investigations are also required on larger samples, on different types of oral ulcers, and on different populations, as well as to identify the potential effects of ozone on different types (major, minor and herpetiform ulcers), frequency and number of RAS lesions. Also, studies are required to measure effects of ozone therapy against or in conjunction with other treatment paradigms used for management of RAS.

## Conclusions

Application of ozone on RAS lesions for 60 seconds lead to a reduction of pain levels as well as enhanced healing of the ulcers by reducing the size and duration of ulcers.

## Additional Information

**How to cite this article**: AL-Omiri, M. K. *et al*. Ozone treatment of recurrent aphthous stomatitis: a double blinded study. *Sci. Rep.* **6**, 27772; doi: 10.1038/srep27772 (2016).

## Figures and Tables

**Table 1:** Distribution of means, standard deviations, minimum and maximum values of recorded ulcer size among study groups (n = 69 for each group).

Recording time	Mean ulcer size in mm (SD)		Minimum ulcer sizein mm		Maximum ulcer sizein mm	
	Test group	Control group	Testgroup	Controlgroup	Testgroup	Controlgroup
Study baseline	5.20 (1.25)	5.25 (1.26)	3.50	3.50	8.00	8.00
Day 1	5.20 (1.25)	5.25 (1.26)	3.50	3.50	8.00	8.00
Day 2	5.12 (1.18)	5.23 (1.23)	3.00	3.50	7.50	8.00
Day 3	4.67 (0.96)	5.21 (1.21)	3.00	3.50	6.50	8.00
Day 4	3.93 (0.87)	5.16 (1.18)	2.50	3.50	5.50	8.00
Day 5	3.10 (0.84)	4.52 (1.14)	2.00	3.00	4.50	7.00
Day 6	1.82 (0.73)	3.91 (1.04)	1.00	2.50	3.00	6.00
Day 7	0.78 (0.47)	2.86 (1.07)	0.00	1.00	1.50	5.00
Day 8	0.27 (0.32)	1.65 (0.94)	0.00	0.00	1.00	3.50
Day 9	0.03 (0.12)	0.52 (0.55)	0.00	0.00	0.50	1.50
Day 10	0.00 (0.00)	0.31 (0.12)	0.00	0.00	0.00	0.50
Day 11	0.00 (0.00)	0.10 (0.11)	0.00	0.00	0.00	0.50
Day 12	0.00 (0.00)	0.04 (0.14)	0.00	0.00	0.00	0.20
Days 12 to15	0.00 (0.00)	0.00 (0.00)	0.00	0.00	0.00	0.00

SD: Standard Deviation.

**Table 2:** Distribution of means, standard deviations, minimum and maximum VAS score values of reported pain levels among study groups (n =  69 for each group).

Recording time	Mean VAS score for pain (SD)		Minimum VAS scorefor pain		Maximum VAS scoreof pain	
	Test group	Control group	Testgroup	Controlgroup	Testgroup	Controlgroup
Study baseline	7.68 (0.81)	7.58 (0.83)	6	6	10	10
Day 1	5.61 (0.97)	7.58 (0.83)	3	6	8	10
Day 2	4.30 (1.06)	7.58 (0.83)	2	6	6	10
Day 3	3.19 (1.20)	6.90 (0.82)	1	5	5	9
Day 4	2.68 (1.05)	6.36 (0.62)	1	5	5	8
Day 5	2.10 (0.86)	5.29 (0.62)	1	4	4	6
Day 6	1.59 (0.60)	4.14 (0.60)	1	3	3	5
Day 7	1.03 (0.54)	2.83 (0.64)	0	1	2	4
Day 8	0.48 (0.53)	1.57 (0.76)	0	0	2	3
Day 9	0.03 (0.17)	0.67 (0.07)	0	0	1	2
Day 10	0.00 (0.00)	0.09 (0.28)	0	0	0	1
Day 11	0.00 (0.00)	0.03 (0.17)	0	0	0	1
Days 12 to 15	0.00 (0.00)	0.00 (0.00)	0	0	0	0

SD: Standard Deviation.

**Table 3:** Correlations between age and gender on one side and each of pain levels, ulcer size and ulcer duration among the study participants (n =  69 for each group).

	Age		Gender	
	Test group	Controlgroup	Test group	Controlgroup
Pain levels				
R=	0.176	0.167	0.02	0.156
P=	0.149	0.170	0.867	0.201
Ulcer size				
R=	0.116	0.178	0.003	0.196
P=	0.341	0.143	0.980	0.107
Ulcer duration				
R=	0.126	0.199	0.043	0.279*
P=	0.303	0.101	0.728	0.020

R =  Pearson’s correlation coefficient; P =  Probability levels; *Significant relation.

**Table 4:** Paired samples t-test to compare differences in ulcer size within each study group.

Group (n = 69for each group)	Ulcer size pairs	Paired Differences			T	df	Sig.(2-tailed)
		Std. ErrorMean	95% ConfidenceInterval of theDifference				
			Lower	Upper			
Test	baseline – Day 2	0.02448	0.03086	0.12856	3.256	68	0.002
	baseline – Day 3	0.05920	0.41086	0.64711	8.936	68	0.000
	baseline – Day 4	0.07045	1.12753	1.40870	17.999	68	0.000
	baseline – Day 5	0.07948	1.93561	2.25280	26.350	68	0.000
	baseline – Day 6	0.09023	3.19675	3.55687	37.423	68	0.000
	baseline – Day 7	0.10923	4.19508	4.63100	40.402	68	0.000
	baseline – Day 8	0.12183	4.68443	5.17064	40.447	68	0.000
	baseline – Day 9	0.14623	4.87486	5.45847	35.332	68	0.000
	baseline – Day 10	0.15144	4.89346	5.49785	34.308	68	0.000
Control	baseline – Day 2	0.01449	−0.01443	0.04341	1.000	68	0.321
	baseline – Day 3	0.01881	−0.00130	0.07376	1.927	68	0.058
	baseline – Day 4	0.02912	0.02885	0.14506	2.986	68	0.004
	baseline – Day 5	0.04198	0.64087	0.80840	17.262	68	0.000
	baseline – Day 6	0.04896	1.24288	1.43827	27.382	68	0.000
	baseline – Day 7	0.06032	2.26369	2.50443	39.523	68	0.000
	baseline – Day 8	0.07022	3.45407	3.73433	51.183	68	0.000
	baseline – Day 9	0.09930	4.52648	4.92279	47.578	68	0.000
	baseline – Day 10	0.14537	4.91281	5.49299	35.790	68	0.000

**Table 5:** Paired samples t-test to compare differences in pain levels within each study group.

Group (n = 69for each group)	Pain level pairs	Paired Differences			T	df	Sig.(2-tailed)
		Std. ErrorMean	95% ConfidenceInterval of theDifference				
			Lower	Upper			
Test	baseline – Day 1	0.10389	1.86516	2.27977	19.949	68	0.000
	baseline – Day 2	0.10136	3.17456	3.57907	33.316	68	0.000
	baseline – Day 3	0.12148	4.25034	4.73517	36.982	68	0.000
	baseline – Day 4	0.10728	4.78593	5.21407	46.607	68	0.000
	baseline – Day 5	0.09326	5.39361	5.76582	59.827	68	0.000
	baseline – Day 6	0.08696	5.91344	6.26048	70.000	68	0.000
	baseline – Day 7	0.08717	6.47823	6.82612	76.313	68	0.000
	baseline – Day 8	0.07608	7.05108	7.35472	94.673	68	0.000
	baseline – Day 9	0.09422	7.46416	7.84019	81.217	68	0.000
	baseline – Day 10	0.09792	7.48577	7.87655	78.446	68	0.000
Control	baseline – Day 3	0.05651	0.56839	0.79393	12.053	68	0.000
	baseline – Day 4	0.07407	1.06960	1.36519	16.437	68	0.000
	baseline – Day 5	0.09023	2.10980	2.46991	25.377	68	0.000
	baseline – Day 6	0.08875	3.25769	3.61188	38.702	68	0.000
	baseline – Day 7	0.07836	4.59725	4.90999	60.662	68	0.000
	baseline – Day 8	0.08385	5.84718	6.18180	71.733	68	0.000
	baseline – Day 9	0.08191	6.74960	7.07649	84.400	68	0.000
	baseline – Day 10	0.08910	7.31496	7.67054	84.097	68	0.000
